# Desmetramadol Is Identified as a G-Protein Biased µ Opioid Receptor Agonist

**DOI:** 10.3389/fphar.2019.01680

**Published:** 2020-01-30

**Authors:** John A. Zebala, Aaron D. Schuler, Stuart J. Kahn, Dean Y. Maeda

**Affiliations:** Department of Chemistry and Preclinical Development, Syntrix Pharmaceuticals, Auburn, WA, United States

**Keywords:** respiratory depression, fentanyl, biased agonists, opioid crisis, CYP2D6, tramadol, O-desmethyltramadol, βarrestin

## Abstract

Tramadol is widely used globally and is the second most prescribed opioid in the United States. It treats moderate to severe pain but lethal opioid-induced respiratory depression is uncommon even in large overdose. It is unknown why tramadol spares respiration. Here we show its active metabolite, desmetramadol, is as effective as morphine, oxycodone and fentanyl in eliciting G protein coupling at the human µ opioid receptor (MOR), but surprisingly, supratherapeutic concentrations spare human MOR-mediated βarrestin2 recruitment thought to mediate lethal opioid-induced respiratory depression.

## Introduction

Mortality due to fatal opioid-induced respiratory depression is the major adverse outcome of the United States (U.S.) opioid crisis (U.S. Senate Committee on Health Education Labor and Pensions, 2018; Volkow and Baler, 2018). Amidst this crisis, prescriber use of tramadol has increased substantially because of its lower risks of abuse (U.S. schedule IV) and fatality in overdose. Tramadol was the second most prescribed opioid in the U.S. in 2017 with 41 million prescriptions compared to only 27 million prescriptions in 2010 (Drug Enforcement Administration, 2018; Centers for Disease Control and Prevention, 2019). During the same period, prescriptions for non-tramadol opioids decreased from 224 million to 155 million. An abuser may suffer fatal respiratory depression from a highly potent schedule II opioid because of an unintentional dosing error of only a few milligrams, or in the case of fentanyl, micrograms (Murphy et al., 2018; U.S. National Library of Medicine, 2018). In contrast, tramadol does not cause significant respiratory depression at therapeutic or supratherapeutic gram doses, and for this reason unintentional fatal dosing errors by abusers are rare (Houmes et al., 1992; Vickers et al., 1992; Tarkkila et al., 1997; Mildh et al., 1999; Grond and Sablotzki, 2004; Ryan and Isbister, 2015). There were no deaths in 71 adults overdosed with tramadol (median dose of 1,000 mg; range: 450-6,000 mg) (Ryan and Isbister, 2015). Of these, 82% (n=58) had no respiratory depression, including a subject who had ingested 6 grams. The remaining 18% (n=13) exhibited only sub-lethal respiratory depression (oxygen saturation <94%), and 5 of these had taken co-ingestants or had pneumonia. Another 114 adult subjects were reported overdosed with up to 14 grams tramadol (mean 1650 mg), or 280-fold the typical 50 mg therapeutic dose (Shadnia et al., 2008). Eight (7%) required ventilation and there were only 2 (2%) fatalities (5 and 8 grams tramadol as the sole intoxicant). Lethal overdose due to tramadol alone is reported to be rare in adults (De Decker et al., 2008; Shadnia et al., 2008; De Backer et al., 2010). Whereas fatal respiratory depression is an uncommon feature of adult tramadol overdose, comparatively little data is available in pediatric subjects who received 167,000 tramadol prescriptions in the U.S. in 2014 (U.S. Food and Drug Administration, 2017). Nine pediatric cases of respiratory depression were reported to the U.S. Food and Drug Administration (FDA) between 1969 and 2016 (U.S. Food and Drug Administration, 2017) and another 15 cases were reported to the World Health Organization between 1992 and 2016 (Rodieux et al., 2018). Dose was unspecified in these 15, and 6 involved other opioids or depressants.

Death certificates are the foundation of drug overdose mortality surveillance in the U.S. However, in multi-drug intoxications which typify overdose mortality, it is not possible to tease out an individual drug's role and thus it is customary to include all drugs from toxicology testing with concentrations greater than trace amounts in the cause-of-death statement (Slavova et al., 2019). The U.S. Centers for Disease Control (CDC) collates and reports these death certificate-listed drugs ‘involved in' overdose deaths and thus reports drugs both causal and not causal to death, with the latter detectable post mortem at least because of common prescribing or availability without prescription (Hedegaard et al., 2018). Among U.S. drug overdose deaths in 2016 (*n* = 63,632), the CDC reported that the abundantly used over-the-counter drug diphenhydramine (Benadryl®) was present in 2,008 overdose deaths (rank 11). Gabapentin was reported present in 1,546 overdose deaths (rank 13), and is among the most prescribed drugs in the U.S. with 64 million prescriptions in 2016 (Goodman and Brett, 2017). Fentanyl and heroin were most common, being present in 18,335 (rank 1) and 15,961 (rank 2) overdose deaths, respectively, but this is probably a significant underestimate given that only 51-75% of medical examiners and/or coroners always request testing for fentanyl and heroin and only 75% always request testing for novel psychoactive substances such as fentanyl analogues (Slavova et al., 2019). Tramadol, with 44 million prescriptions in 2016, was reported by the CDC to be present in 1,250 overdose deaths, ranking below gabapentin and diphenhydramine; 266 had intent of suicide and drugs co-present with tramadol were undisclosed. Normalizing fatality to the amount of drug dispensed, tramadol remained below gabapentin in rank and exhibited the lowest rate of fatality in the U.S. of any marketed opioid (<0.01 death per 100,000 grams dispensed) (Murphy et al., 2018).

**Figure 1:**
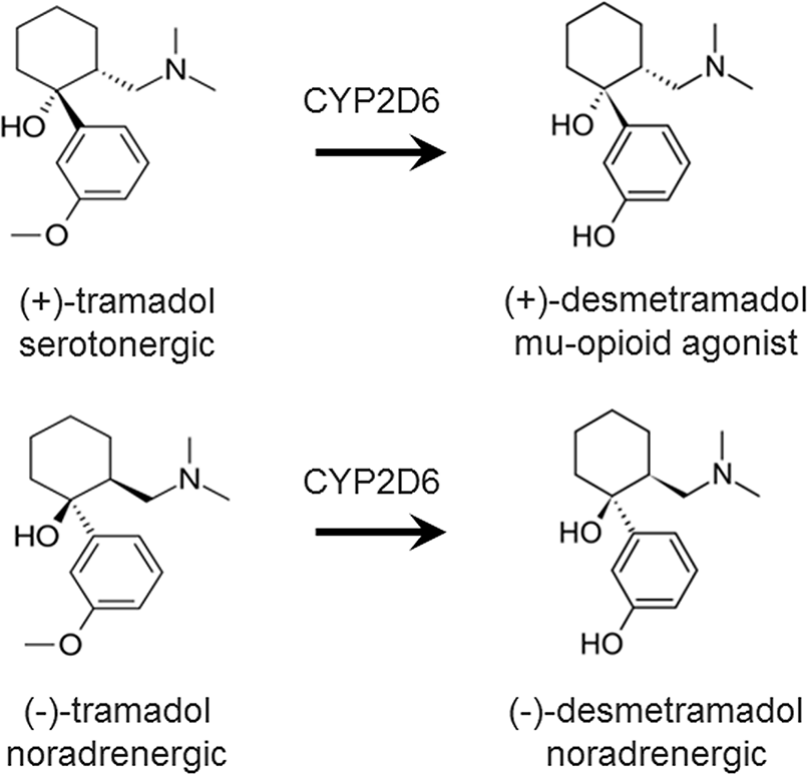
Chemical structure and dominant pharmacology of each enantiomer of tramadol and desmetramadol. Desmetramadol is also known as M1 or O-desmethyltramadol. CYP2D6, cytochrome P450 isozyme 2D6.

Tramadol is racemic and its clinical efficacy requires metabolism by CYP2D6 to its racemic active metabolite, desmetramadol (**Figure 1**) (Grond and Sablotzki, 2004). Tramadol analgesia arises from a combination of norepinephrine reuptake inhibition (NRI) by the negative enantiomers of tramadol and desmetramadol, and MOR agonism by (+)-desmetramadol. Abnormal tramadol metabolism (too low or too high) due to co-ingestion of a CYP2D6 inhibitor (drug-drug interaction) or genetics (CYP2D6 gene defects or duplications) is a common occurrence in clinical practice affecting over a third to a half of patients that gives rise to inadequate efficacy or increased adverse events (Zebala et al., 2019). The FDA amended the tramadol label in 2017 with warnings reflecting its metabolic liabilities that affect its efficacy and safety in adult and pediatric patients that included various contradictions on its use in children and a warning that patients of any age who are CYP2D6 ultra-rapid metabolizers (>2 genes) should not use tramadol (U.S. Food and Drug Administration, 2017). We have sought to overcome these liabilities by advancing desmetramadol into the clinic as a strategy to provide NRI and MOR agonism without metabolism. We demonstrated desmetramadol provides the same human analgesic and safety profile as tramadol, but without its metabolic liabilities (Zebala et al., 2019).

Despite tramadol's decades-long clinical use it is not understood why it spares respiration in humans. The positive (+) desmetramadol enantiomer has only 5- to 7-fold lower affinity and efficacy for the human MOR as morphine ([^3^H]naloxone competition: K_i_ of 3.4 vs. 0.62 nM; [^35^S]GTPγS binding: EC_50_ of 860 vs. 118 nM and E_max_ of 52% for both, where E_max_ of 100% defined as response for the enkephalin analog, [D-Ala^2^, NMe-Phe^4^, Gly-ol^5^]-enkephalin, or “DAMGO”) (Gillen et al., 2000). Blood desmetramadol exceeds its human MOR K_i_ by 130-fold at the maximum therapeutic tramadol dose and by as much as 3,000-fold at a lethal tramadol dose (Grond and Sablotzki, 2004; De Backer et al., 2010). Because blood desmetramadol is proportional to tramadol dose from therapeutic to lethal doses, the sparing of respiratory depression by tramadol cannot be attributed to metabolism, which offers no protective saturable ceiling on systemic exposure to desmetramadol.

The MOR is a G protein-coupled receptor (GPCR) that mediates two distinct downstream intracellular signaling pathways: (i) activating the Gα_i_ subunit, which then inhibits cyclic AMP (cAMP) levels, and (ii) recruiting βarrestins, scaffolding proteins that regulate subsequent GPCR signaling. Gene knock-out studies have shown that activation of the MOR separately mediates analgesia through G protein signaling, and respiratory depression through βarrestin2 recruitment (Raehal et al., 2005). "Biased" GPCR ligands induce alternative receptor conformations that can selectively activate one intracellular signaling pathway over another. Fentanyl is an example of a βarrestin2-biased MOR ligand that causes more respiratory depression than morphine at equianalgesic doses (Schmid et al., 2017). Conversely, a MOR agonist with bias for G-protein signaling over βarrestin2 recruitment provides a lower level of respiratory depression for a given level of analgesia, and is a strategy for developing safer opioids (Schmid et al., 2017; Viscusi, 2019). Other studies suggest the possibility that the lower level of respiratory depression observed for such G-protein biased agonists may be due to bias at other levels of MOR signaling (Kliewer et al., 2019).

The potent affinity of (+)-desmetramadol for the human MOR and the clinical properties of tramadol prompted us to hypothesize that G protein-biased MOR agonism by desmetramadol might account for the apparent discrepant potent MOR binding by desmetramadol and the empirical sparing of lethal respiratory depression in tramadol overdose. Here we investigate this hypothesis by using cell-based assays to measure human MOR-mediated G protein signaling and βarrestin2 recruitment by desmetramadol, each of its enantiomers, and clinically relevant control opioids having moderate (morphine, oxycodone) and high (fentanyl) propensity for clinical respiratory depression.

## Methods

### Opioid Agonists

Morphine, oxycodone, and fentanyl were obtained from Cerilliant (Round Rock, TX, USA) and were evaporated and reconstituted in DMSO. Desmetramadol and its enantiomers were chemically synthesized (**Supplementary Methods**). Agonists were serially diluted 3-fold in DMSO (morphine, oxycodone, fentanyl) or water (desmetramadol and its enantiomers) to concentrations 100-fold the assay test concentration (i.e., the DMSO or water vehicle was 1% in the final assay). Further dilution with assay buffer, OptiMEM® + 0.1% BSA or HBSS + 10 mM HEPES, provided samples with concentrations 5-fold or 4-fold the assay test concentration, respectively.

### cAMP Accumulation and βarrestin2 Recruitment

To determine cAMP accumulation and βarrestin2 recruitment by the human MOR, commercial enzyme fragment complementation assays (β-galactosidase) were used (Eurofins-DiscoverX, Fremont, California). PathHunter® CHO-K1 OPRM1 β-Arrestin cells were plated at a density of 5,000 cells per well of a 384-well, white-walled assay microplate in Assay Complete Cell Plating 2 Reagent (DiscoverX) overnight prior to measuring the signal. Cells were treated for 90-180 min with agonist at 37 ^o^C and signal determined using the PathHunter Detection Kit to detect functional β-galactosidase. Test concentrations were established in each well with the 5-fold more concentrated samples, and were serial 3-fold reductions of the largest test concentration (100 µM). The resulting increase in luminescence was measured using an Envision^TM^ (PerkinElmer) microplate reader in relative light units (RLU). The control agonist is [Met]-enkephalin. Percentage activity is computed as 100% x (test agonist mean RLU - vehicle control mean RLU)/(control agonist maximum RLU – vehicle control mean RLU). Maximum control agonist RLU is 24-fold over vehicle control RLU.

cAMP Hunter™ CHO-K1 OPRM1 G_i_ cells were plated at a density of 10,000 cells per well of a 384-well and incubated as described for βarrestin2 recruitment. Cells were then stimulated for 30 min at 37 ^o^C with agonist and 20 µM forskolin, or with 20 µM forskolin only. The same agonist test concentrations were employed as described for βarrestin2 recruitment. Test concentrations were established in each well with the 4-fold more concentrated samples. Following stimulation, signal was detected using the HitHunter cAMP Assay XS+ Detection Kit and luminescence measured as described for βarrestin2 recruitment. Maximum control agonist RLU is 13-fold over vehicle control RLU.

### Software and Analysis

GraphPad Prism software (v. 8.0) was used for data analyses. Agonists were assayed in duplicate. Concentration response data were fit to a four-parameter non-linear regression model to determine EC_50_ and E_max_, with the mean ± standard deviation (SD) of the values from each experiment reported.

## Results

Commercially available enzyme fragment complementation assays were used to assess each agonist's potency (EC_50_) and efficacy (E_max_) of G-protein modulated cAMP accumulation and βarrestin2 recruitment (**Table 1**). The potency of (+)-desmetramadol was comparable to oxycodone (EC_50_ mean [SD] = 63 [4] vs 67 [10] nM) and 38-fold greater than the negative desmetramadol enantiomer. Desmetramadol (racemic) was half as potent as the positive enantiomer in keeping with the relative potencies of each enantiomer and each present in equal amount. Morphine induced G-protein modulated cAMP signaling with 20-fold greater potency than (+)-desmetramadol (EC_50_ mean [SD] = 3 [0.4] vs 63 [4] nM) consistent with their reported relative affinity for the human MOR. Desmetramadol, its enantiomers, and all controls performed as full agonists in the cAMP assay for G-protein signaling (each E_max_ 100% of morphine).

**Table 1:** Human MOR-mediated G-protein modulated cAMP and βarrestin2 recruitment by control agonists (morphine, oxycodone, fentanyl), desmetramadol and its enantiomers.

Agonist	cAMP		βarrestin2		Agonist:Morphine^b^	
	EC_50_ (nM)	E_max_ (%)	EC_50_ (nM)	E_max_ (%)	cAMP E_max_	βarrestin2 E_max_
Morphine	3 ± 0.4	100 ± 0.2	352 ± 11	69 ± 0.04	1.0	1.0
Oxycodone	67 ± 10	100 ± 0.6	2233 ± 99	66 ± 2	1.0	0.96
Fentanyl	0.13 ± 0.007	100 ± 0.4	35 ± 1	151 ± 0.6	1.0	2.2***
Desmetramadol	110 ± 9	101 ± 0.6	3821 ± 223	19 ± 0.3	1.0	0.28***
(-)-Desmetramadol	2426 ± 469	100 ± 2.6	>100,000	15 ± 2^a^	1.0	0.22**
(+)-Desmetramadol	63 ± 4	99 ± 0.9	2672 ± 733	27 ± 4	1.0	0.39**

Potency (EC_50_) and efficacy (E_max_, expressed as percent of [Met]-enkephalin) values determined by cAMP and βarrestin2 assays with cAMP Hunter™ CHO-K1 OPRM1 G_i_ and PathHunter® CHO-K1 OPRM1 β-Arrestin cell lines, respectively. Data are mean ± SD of assays run in duplicate. Maximum stimulation was tested at 100 µM concentration to establish E_max_ for each agonist.

a Value at 100 µM concentration presented rather than E_max_ because dose-response curve did not plateau.

b Unpaired, two-tailed *t* test of agonist E_max_ versus morphine E_max_. **P < 0.005, ***P < 0.0001.

Consistent with the findings of other investigators, the maximum βarrestin2 recruitment by fentanyl was significantly greater than the maximum βarrestin2 recruitment by morphine (E_max_ 220% of morphine, *P* < 0.0001; i.e., βarrestin2-biased). In contrast, both desmetramadol and its enantiomers significantly spared human MOR-mediated βarrestin2 recruitment up to the largest concentration tested (100,000 nM), with desmetramadol plateauing to a significantly lower maximum βarrestin2 recruitment compared to morphine (E_max_ 28% of morphine, *P* < 0.0001). The maximum βarrestin2 recruitment by desmetramadol was only 13% that of fentanyl.

**Figure 2:**
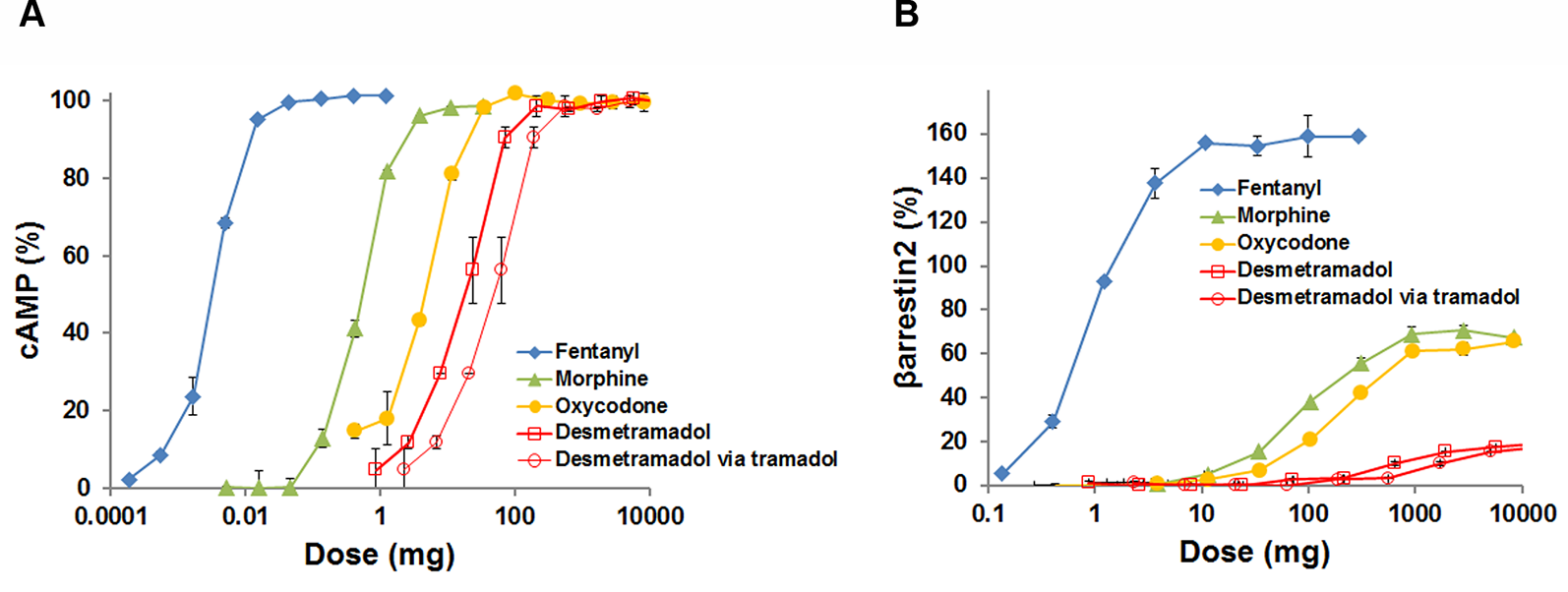
G-protein modulated cAMP and βarrestin2 recruitment by the human MOR as a function of human dose. The adult human dose-to-maximum blood concentration (C_max_) relationship for each opioid was used to model maximum cAMP accumulation and βarrestin2 recruitment as a function of human dose, where Dose = Reference Dose x (C_tested_/C_max_) and the Reference Dose (mg) and C_max_ (µM) are from **Supplementary Table 1** and C_tested_ (µM) is the tested agonist concentration. Data are means ± SD, displayed as percent of maximum [Met]-enkephalin efficacy for assays run in duplicate. Data for doses >10 grams and > E_max_ were censored for clinical irrelevance and to permit visualization of each curve, respectively. **(A)** Percent cAMP versus dose. **(B)** Percent βarrestin2 recruitment versus dose. Data for desmetramadol are shown for both direct dosing of desmetramadol and indirect dosing of desmetramadol via tramadol, its prodrug.

To place these *in vitro* data in the context of dose escalation in the setting of opioid abuse, human pharmacokinetics for each agonist (**Supplementary Table 1**) were used to map each tested agonist concentration (C_tested_) to a proportional adult human dose having a maximum blood concentration (C_max_) equal to C_tested_. The C_tested_-to-dose model approximates the maximum cAMP response and βarrestin2 recruitment *in vivo* as a function of human dose (**Figure 2**). The model shows that clinically analgesic doses of fentanyl (0.050-0.100 mg), morphine (10–20 mg) and oxycodone (15–30 mg) all correspond to a maximum cAMP response on the plateau of each curve (**Figure 2A**). Clinically analgesic doses of desmetramadol (20–30 mg) and tramadol (50–100 mg) correspond to lower cAMP responses (~50%) consistent with their known MOR-mediated and non-MOR analgesic mechanisms. The same analgesic doses correspond to βarrestin2 recruitment ranging from none for desmetramadol and tramadol, to modest recruitment (<10%) for morphine, oxycodone and fentanyl (**Figure 2B**). Lethal doses of morphine (>300 mg), oxycodone (>200 mg) and fentanyl (>1–2 mg) correspond to βarrestin2 recruitment >40%. In contrast, βarrestin2 recruitment by tramadol and desmetramadol is comparatively spared, corresponding to a maximum of 10–18% for doses between 1 and 10 g.

## Discussion

Our data show desmetramadol, tramadol's active metabolite, to be a potent G-protein biased human MOR agonist. It elicits maximum human MOR-mediated G protein coupling thought to mediate analgesia as effectively as morphine, oxycodone and fentanyl, with the positive enantiomer having a potency for cAMP accumulation equal to oxycodone. The positive enantiomer was shown by Gillen et al. (2000) to have 7-fold lower efficacy than morphine for GTPγS binding. Clinical studies show that orally administered tramadol provides analgesia equal to morphine (Grond and Sablotzki, 2004). These data are at odds with the perception that tramadol is a “weak” opioid, either in terms of its analgesic efficacy or as an explanation for why it spares respiration. This perception more likely is the result of its inconsistent metabolism to the active metabolite, which we have shown is obviated by dosing desmetramadol directly (Zebala et al., 2019).

Instead, we observed that desmetramadol spares MOR-mediated βarrestin2 recruitment thought to mediate opioid-induced respiratory depression, and this may explain the observed clinical sparing of lethal respiratory depression by tramadol. Whereas our data indicate no βarrestin2 recruitment was associated with therapeutic doses of desmetramadol and tramadol, therapeutic doses of morphine and oxycodone corresponded to a modest level of βarrestin2 recruitment. Consistent with these data, clinical studies that compared tramadol to morphine, oxycodone and meperidine (pethidine) for treating moderate to severe pain found that all opioids except tramadol elicited respiratory depression at equianalgesic doses (Houmes et al., 1992; Tarkkila et al., 1997; Mildh et al., 1999).

Our data indicate that lethal human doses of morphine, oxycodone and fentanyl correspond to levels of βarrestin2 recruitment exceeding 40%, or about 4-fold the level associated with therapeutic doses. In contrast, βarrestin2 recruitment by tramadol and desmetramadol plateaued at a significantly lower level of βarrestin2 recruitment, reaching a maximum of 10–18% only for large doses corresponding to 1 to 10 grams, a level of βarrestin2 recruitment nearly equal to that achieved by oxycodone and morphine at their therapeutic doses. As with other pharmacodynamic phenomena that exhibit a dose-response relationship, these data suggest clinical lethality due to opioid-induced respiratory depression is a threshold phenomenon. In this threshold model, increasing βarrestin2 recruitment is associated with increasing sublethal respiratory depression as a function of increasing dose, until a critical lethal threshold is crossed. If a biased opioid agonist increases βarrestin2 recruitment, but cannot cross this threshold for lethality even in large overdose (i.e., βarrestin2 recruitment is capped), it may decrease respiration up to a certain level, but will have a low propensity to induce a lethal level of respiratory depression by itself. Illicit desmetramadol was reported lethal at blood concentrations consistent with gram doses, but correlating dose to fatality is obscured in these cases because desmetramadol was coingested with other opioids and depressants that included mitragynine, benzodiazepines and ethanol that could have reduced the lethal βarrestin2 threshold (Kronstrand et al., 2011). Lethality due to tramadol alone however, requires ingestion of at least 100 times the therapeutic 50 mg dose (i.e., 100 standard 50 mg tablets), and effects of its intoxication reflect more its monoaminergic activity and seizures, rather than MOR agonist-induced respiratory depression (De Decker et al., 2008; Shadnia et al., 2008; De Backer et al., 2010).

Biased signaling by a MOR agonist can be quantified by calculating a bias factor that compares βarrestin2 recruitment to either GTPγS binding or inhibition of cAMP accumulation by the agonist relative to a reference agonist (Winpenny et al., 2016). As observed for other biased MOR agonists including fentanyl (Schmid et al., 2017), we found that fentanyl, desmetramadol and its enantiomers more reliably computed as G-protein biased by comparing βarrestin2 recruitment with GTPγS binding rather than to inhibition of cAMP accumulation (**Supplementary Table 2**).

There are limitations to using only the bias factor to identify a safer opioid. The bias factor only defines the extent of a normalized difference, or window between the two dose-response curves for G protein and βarrestin2 signaling. It does not provide relevant clinical context in the setting of abuse, where lethality turns on a human subject achieving a lethal threshold of βarrestin2 signaling by intentional dose escalation irrespective of its separation from G-protein signaling. An opioid with an impressive bias factor will still be prone to lethality in the setting of abuse if it elicits full βarrestin2 signaling at doses readily reached by intentional or unintentional dose escalation. In the threshold model, the clinically relevant measure of opioid safety is whether βarrestin2 recruitment is capped below a lethal threshold for any dose that is practical to intentionally or unintentionally administer in the setting of abuse (rationalizing our mapping each C_tested_ to a human dose and C_max_).

In this regard, the maximum degree to which desmetramadol spares βarrestin2 recruitment relative to morphine is comparable to other G-protein biased MOR agonists, including the clinically tested oliceridine (**Table 2**) (Dewire et al., 2013; Schmid et al., 2017). βarrestin2 recruitment corresponding to therapeutic blood concentrations of morphine and oliceridine ranged from indistinguishable to only minimally different from one another (**Supplementary Figure 1**). This may explain why trials of therapeutic doses of morphine and oliceridine variably demonstrated statistically significant and insignificant differences in sublethal respiratory depression (Viscusi, 2019). Unlike multi-gram tramadol overdose, human subjects have not been exposed to oliceridine doses that would cause much larger differences in βarrestin2 recruitment compared to a lethal morphine dose.

**Table 2:** βarrestin2 recruitment efficacy by desmetramadol and clinical (oliceridine) and preclinical (SR-15098, SR-15099, SR-17018) biased agonists.

Study	MOR Agonist	βarrestin2^a^ E_max_ (%)	Agonist:Morphine βarrestin2 E_max_	Agonist:Fentanyl βarrestin2 E_max_
This study				
	Morphine	69		
	Fentanyl	151		
	Desmetramadol	19^b^	0.28	0.13
				
Dewire et al. (2013)				
	Morphine	99		
	Fentanyl	478		
	Oliceridine (TRV130)	14^c^	0.14	0.03
				
Schmid et al. (2017)				
	Morphine	24		
	Fentanyl	60		
	SR-15098	12^d^	0.50	0.20
	SR-15099	3^d^	0.13	0.05
	SR-17018	10^d^	0.42	0.17

a Mean βarrestin2 recruitment efficacy (E_max_) at human MOR as percent of[Met]-enkephalin, morphine and [D-Ala^2^, NMe-Phe^4^, Gly-ol^5^]-enkephalin (DAMGO) in this study, Dewire et al. (2013) and Schmid et al. (2017), respectively.

b Maximum stimulation tested at 100 µM concentration to establish E_max_.

c Maximum stimulation tested at 6.6 µM concentration to establish E_max_.

d Stimulation at 10 µM maximum concentration presented rather than E_max_ because dose-response curve did not plateau.

Decades of tramadol use has resulted in >30 billion cumulative patient treatment days worldwide, and by way of its active metabolite may have validated biased agonism as a strategy for safer opioids (Grünenthal Gmbh, 2017). Our results combined with empirical clinical evidence suggest that the absence of respiratory depression by tramadol correlates to sparing of βarrestin2 recruitment by its active metabolite, desmetramadol, newly identified herein as a potent G-protein biased human MOR agonist.

## Data Availability Statement

The datasets generated for this study are available on request to the corresponding author.

## Author Contributions

JZ conceptualized the study and drafted the manuscript with support from DM and SK. AS synthesized desmetramadol enantiomers.

## Funding

This work was supported by USPHS grant DA027304 from the National Institute on Drug Abuse. All authors are employees at Syntrix Pharmaceuticals.

## Conflict of Interest

All authors are employees at Syntrix Pharmaceuticals.

## References

[B1] Centers for Disease Control and Prevention (2019). U.S. Opioid Prescribing Rate Maps. United States Centers for Disease Control and Prevention. Available: https://www.cdc.gov/drugoverdose/maps/rxrate-maps.html [Accessed October 18 2019]

[B2] De Backer B. Renardy F. Denooz R. Charlier C. (2010). Quantification in postmortem blood and identification in urine of tramadol and its two main metabolites in two cases of lethal tramadol intoxication. J. Anal. Toxicol. 34, 599–604. 10.1093/jat/34.9.599 21073815

[B3] De Decker K. Cordonnier J. Jacobs W. Coucke V. Schepens P. Jorens P. G. (2008). Fatal intoxication due to tramadol alone: case report and review of the literature. Forensic. Sci. Int. 175, 79–82. 10.1016/j.forsciint.2007.07.010 17875377

[B4] Dewire S. M. Yamashita D. S. Rominger D. H. Liu G. Cowan C. L. Graczyk T. M. (2013). A G protein-biased ligand at the mu-opioid receptor is potently analgesic with reduced gastrointestinal and respiratory dysfunction compared with morphine. J. Pharmacol. Exp. Ther 344, 708–717. 10.1124/jpet.112.201616 23300227

[B5] Drug Enforcement Administration (2018). Tramadol (Trade Names: Ultram®, Ultracet®). United States Department of Justice, Drug Enforcement Administration. Available: https://www.deadiversion.usdoj.gov/drug_chem_info/tramadol.pdf [Accessed November, 5 2018]

[B6] Gillen C. Haurand M. Kobelt D. J. Wnendt S. (2000). Affinity, potency and efficacy of tramadol and its metabolites at the cloned human mu-opioid receptor. Naunyn. Schmiedebergs. Arch. Pharmacol. 362, 116–121. 10.1007/s002100000266 10961373

[B7] Goodman C. W. Brett A. S. (2017). Gabapentin and Pregabalin for Pain - Is Increased Prescribing a Cause for Concern? N. Engl. J. Med. 377, 411–414. 10.1056/NEJMp1704633 28767350

[B8] Grünenthal Gmbh (2017). Comments to the application for inclusion of tramadol into the WHO Model List of Essential Medicines (EML). World Health Organization. Available: https://www.who.int/selection_medicines/committees/expert/21/applications/Grunethal_tramadol.pdf [Accessed July 26 2019]

[B9] Grond S. Sablotzki A. (2004). Clinical pharmacology of tramadol. Clin. Pharmacokinet. 43, 879–923. 10.2165/00003088-200443130-00004 15509185

[B10] Hedegaard H. Bastian B. A. Trinidad J. P. Spencer M. Warner M. (2018). Drugs Most Frequently Involved in Drug Overdose Deaths: United States, 2011-2016. Natl. Vital. Stat. Rep. 67, 1–14. 30707673

[B11] Houmes R. J. Voets M. A. Verkaaik A. Erdmann W. Lachmann B. (1992). Efficacy and safety of tramadol versus morphine for moderate and severe postoperative pain with special regard to respiratory depression. Anesth. Analg. 74, 510–514. 10.1213/00000539-199204000-00007 1554117

[B12] Kliewer A. Schmiedel F. Sianati S. Bailey A. Bateman J. T. Levitt E. S. (2019). Phosphorylation-deficient G-protein-biased mu-opioid receptors improve analgesia and diminish tolerance but worsen opioid side effects. Nat. Commun. 10, 367. 10.1038/s41467-018-08162-1 30664663 PMC6341117

[B13] Kronstrand R. Roman M. Thelander G. Eriksson A. (2011). Unintentional fatal intoxications with mitragynine and O-desmethyltramadol from the herbal blend Krypton. J. Anal. Toxicol. 35, 242–247. 10.1093/anatox/35.4.242 21513619

[B14] Mildh L. H. Leino K. A. Kirvela O. A. (1999). Effects of tramadol and meperidine on respiration, plasma catecholamine concentrations, and hemodynamics. J. Clin. Anesth. 11, 310–316. 10.1016/S0952-8180(99)00047-1 10470633

[B15] Murphy D. L. Lebin J. A. Severtson S. G. Olsen H. A. Dasgupta N. Dart R. C. (2018). Comparative Rates of Mortality and Serious Adverse Effects Among Commonly Prescribed Opioid Analgesics. Drug. Saf. 41, 787–795. 10.1007/s40264-018-0660-4 29582394

[B16] Raehal K. M. Walker J. K. Bohn L. M. (2005). Morphine side effects in beta-arrestin 2 knockout mice. J. Pharmacol. Exp. Ther. 314, 1195–1201. 10.1124/jpet.105.087254 15917400

[B17] Rodieux F. Vutskits L. Posfay-Barbe K. M. Habre W. Piguet V. Desmeules J. A. (2018). When the Safe Alternative Is Not That Safe: Tramadol Prescribing in Children. Front. Pharmacol. 9, 148. 10.3389/fphar.2018.00148 29556194 PMC5844975

[B18] Ryan N. M. Isbister G. K. (2015). Tramadol overdose causes seizures and respiratory depression but serotonin toxicity appears unlikely. Clin. Toxicol. (Phila) 53, 545–550. 10.3109/15563650.2015.1036279 25901965

[B19] Schmid C. L. Kennedy N. M. Ross N. C. Lovell K. M. Yue Z. Morgenweck J. (2017). Bias factor and therapeutic window correlate to predict safer opioid analgesics. Cell. 171 1165-1175, e1113. 10.1016/j.cell.2017.10.035 PMC5731250 29149605

[B20] Shadnia S. Soltaninejad K. Heydari K. Sasanian G. Abdollahi M. (2008). Tramadol intoxication: a review of 114 cases. Hum. Exp. Toxicol. 27, 201–205. 10.1177/0960327108090270 18650251

[B21] Slavova S. Delcher C. Buchanich J. M. Bunn T. L. Goldberger B. A. Costich J. F. (2019). Methodological Complexities in Quantifying Rates of Fatal Opioid-Related Overdose. Curr. Epidemiol. Rep. 6, 263–274. 10.1007/s40471-019-00201-9 31259141 PMC6559129

[B22] Tarkkila P. Tuominen M. Lindgren L. (1997). Comparison of respiratory effects of tramadol and oxycodone. J. Clin. Anesth. 9, 582–585. 10.1016/S0952-8180(97)00147-5 9347436

[B23] U.S. National Library of Medicine (2018). TOXNET. Toxicology Data Network[Online]. United States National Library of Medicine. Available: https://toxnet.nlm.nih.gov [Accessed].

[B24] U.S. Senate Committee on Health Education Labor and Pensions (2018). The Economic Cost of the Opioid Epidemic in Washington State. United States Senate Committee on Health Education Labor and Pensions. Available: https://www.murray.senate.gov/public/index.cfm/2018/4/opioid-crisis-senator-murray-unveils-new-analysis-showing-opioid-crisis-costs-washington-state-billions [Accessed Oct 19 2019]

[B25] U.S. Food and Drug Administration (2017). FDA restricts use of prescription codeine pain and cough medicines and tramadol pain medicines in children; recommends against use in breastfeeding women. United States Food and Drug Administration. Available: https://www.fda.gov/drugs/drug-safety-and-availability/fda-drug-safety-communication-fda-restricts-use-prescription-codeine-pain-and-cough-medicines-and [Accessed December 14 2019]

[B26] Vickers M. D. O'flaherty D. Szekely S. M. Read M. Yoshizumi J. (1992). Tramadol: pain relief by an opioid without depression of respiration. Anaesthesia 47, 291–296. 10.1111/j.1365-2044.1992.tb02166.x 1519677

[B27] Viscusi E. R. (2019). Improving the therapeutic window of conventional opioids: novel differential signaling modulators. Reg. Anesth. Pain. Med. 44, 32–37. 10.1136/rapm-2018-000010 30640650

[B28] Volkow N. D. Baler R. (2018). A prescription for better opioid prescribing? Informing physicians who prescribe opioids about opioid-linked deaths in their practice reduces future opioid prescribing. Nat. Med. 24, 1492–1498. 10.1038/s41591-018-0214-4 30297898

[B29] Winpenny D. Clark M. Cawkill D. (2016). Biased ligand quantification in drug discovery: from theory to high throughput screening to identify new biased mu opioid receptor agonists. Br. J. Pharmacol. 173, 1393–1403. 10.1111/bph.13441 26791140 PMC4940816

[B30] Zebala J. A. Searle S. L. Webster L. R. Johnson M. S. Schuler A. D. Maeda D. Y. (2019). Desmetramadol has the safety and analgesic profile of tramadol without its metabolic liabilities: consecutive randomized, double-blind, placebo- and active comparator-controlled trials. J. Pain. 20, 1218–1235. 10.1016/j.jpain.2019.04.005 31005596 PMC6790288

