# Supplementary material for: Desmetramadol Is Identified as a G-Protein Biased µ Opioid Receptor Agonist
Source: Front Pharmacol. 2020 Jan 30;10:1680. doi: 10.3389/fphar.2019.01680 (PMC7025522; doi:10.3389/fphar.2019.01680)
Supplement: Supplementary file 1 [file DataSheet_1.pdf]

Supplementary Material

Supplementary Methods

Chemical synthesis of desmetramadol and its enantiomers. Diastereomeric salt formation with tramadol hydrochloride (HCl) using L-(-)-di-*p*-toluoyl-tartaric acid and D-(+)-di-*p*-toluoyl-tartaric acid was used to isolate the HCl salt of (-)-tramadol and (+)-tramadol, respectively (Evans, 2001b;a). The analytical data obtained by ^1H NMR, liquid chromatography-mass spectroscopy (LC-MS), elemental analysis and optical rotation for each HCl salt conformed to previously reported values (Buschmann et al., 1998a). Tramadol, (+)-tramadol, and (-)-tramadol were demethylated by diisobutylaluminium hydride (DIBAL) treatment in anhydrous toluene to yield the HCl salt of desmetramadol, (+)-desmetramadol, and (-)-desmetramadol, respectively (Senanayake et al., 2004). The analytical data obtained by ^1H NMR, LC-MS, elemental analysis and optical rotation for each HCl salt of desmetramadol and its enantiomers conformed to previously reported values (Buschmann et al., 1998b). Each tramadol and desmetramadol enantiomer was isolated in 100% enantiomeric purity (enantiomeric excess) as determined by chiral high-pressure liquid chromatography.

Supplementary Table 1. Human dose-to-blood concentration relationships of control opioid agonists, olicerdine and desmetramadol directly dosed and indirectly dosed via tramadol.

Agonist	Reference Dose (mg)	C_{max} (ng/mL)	Agonist MW (g/mole)	C_{max} (µM)
Morphine [a]	20	23	285.34	0.080
Oxycodone [b]	9.1	34	315.36	0.11
Fentanyl [c]	0.050	0.63	336.47	0.0019
Oliceridine [d]	1.5, 4.5	47, 119	386.55	0.12, 0.31
Desmetramadol [e]	20	29	249.35	0.12
Desmetramadol via tramadol [f]	100	55	249.35	0.22

MW, molecular weight; C_{max}, maximum blood concentration.

[a] Single oral administration of immediate-release formulation to adults (*n* = 303) (Collins et al., 1998).

[b] Single oral administration of immediate-release formulation to adults (*n* = 12) (Leow et al., 1992).

[c] Intravenous administration of citrate salt (*n* = 24) (Rauck et al., 2017).

[d] Intravenous administration (*n* = 30) (Soergel et al., 2014).

[e] Single oral administration provides C_{max} half the mean steady-state level for both enantiomers combined (*n* = 43) (Zebala, 2017;Zebala et al., 2019).

[f] Single oral administration of immediate-release formulation to adults (*n* = 18) (Grond and Sablotzki, 2004).

Supplementary Table 2. Bias factors computed from GTPγS binding and cAMP signaling at the human MOR.

	$\Delta\log(E_{max}/EC_{50})$			$\Delta\Delta\log(E_{max}/EC_{50})$	
Agonist	GTPγS	cAMP	βarr2	GTPγS - βarr2 (Bias factor, Bias)	cAMP - βarr2 (Bias factor, Bias)
Morphine	0.00	0.00	0.00	0.00 (1.0, Unbiased)	0.00 (1.0, Unbiased)
Fentanyl	0.11	1.36	1.35	-1.24 (0.1, Arrestin)	0.02 (1.0, Unbiased)
(+)-Desmetramadol	-0.86	-1.33	-1.29	0.43 (2.7, G protein**)	0.03 (1.0, Unbiased)
(-)-Desmetramadol*	-2.74	-2.91	-3.12	0.38 (2.4, G protein)	0.21 (1.6, G protein)
Desmetramadol	-	-1.56	-1.59	-	0.03 (1.1, G protein)
Oliceridine	-	0.72	0.25	-	0.47 (2.9, G protein)

$\Delta\log(E_{max}/EC_{50})$ is a validated alternative to the transduction coefficient ($\Delta\Delta\log(\tau/K_A)$) employed in the operational model of agonism for calculating pathway bias towards $G\alpha_i$-dependent signaling (Winpenny et al., 2016). The displayed $\Delta\log(E_{max}/EC_{50})$ values were computed from the potency (EC_{50}) and efficacy (E_{max}) values obtained across functional assays using values for morphine as the reference agonist. EC_{50} and E_{max} values for GTPγS binding were determined in membranes from CHO-K1 cells transfected with the human MOR as described (Gillen et al., 2000). EC_{50} and E_{max} values for cAMP and βarrestin2 (βarr2) were determined as described herein for fentanyl, desmetramadol, and each of its enantiomers with cAMP Hunter™ CHO-K1 OPRM1 G_i and PathHunter® CHO-K1 OPRM1 β-Arrestin cell lines, respectively. EC_{50} and E_{max} values for cAMP and βarrestin2 were determined for oliceridine as described (DeWire et al., 2013). A bias factor >1 indicates greater bias for G-protein signaling compared to morphine, and a bias factor <1 indicates greater bias for βarrestin2 recruitment. *EC_{50} and E_{max} values used were those values obtained at the maximum tested concentration because the dose-response curve did not plateau. ** Excess receptor reserve in the GTPγS binding assay is ruled out as a specious explanation for the bias because (+)-desmetramadol shows a high intrinsic efficacy (ε=1.23) that was between the intrinsic efficacy (ε) of morphine (ε=1.17) and fentanyl (ε=1.25), but below DAMGO (ε=2.33) (Gillen et al., 2000).

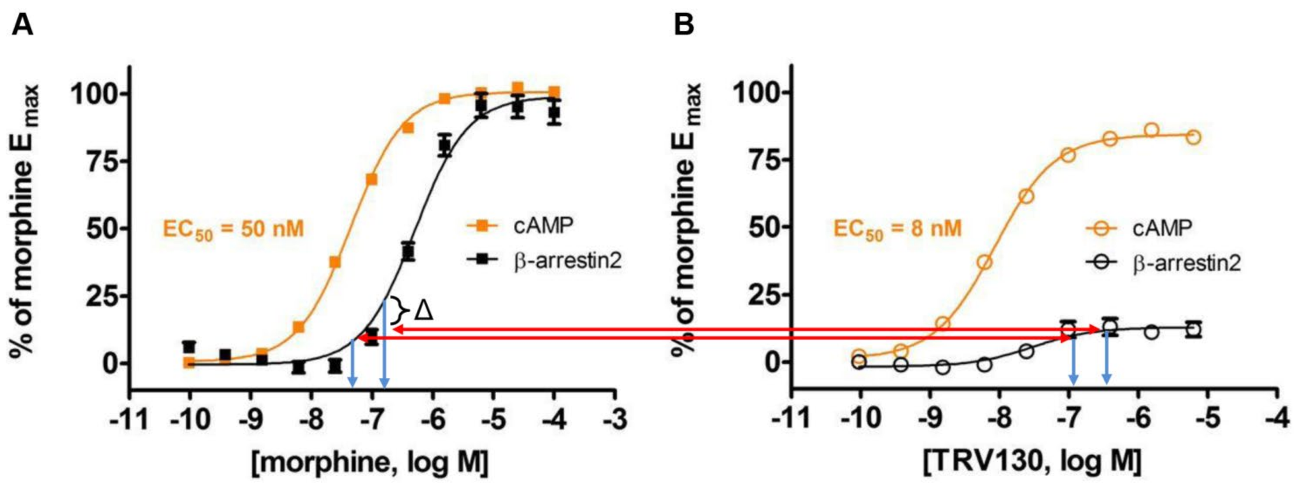

SUPPLEMENTARY FIGURE 1 | Human MOR-mediated G-protein modulated cAMP and βarrestin2 recruitment by morphine and oliceridine (TRV130). Figure adapted from DeWire et al. with permission (DeWire et al., 2013). Data are means ± standard error, displayed as percent of maximum morphine efficacy. **A**, Percent cAMP and βarrestin2 recruitment versus morphine concentration. **B**, Percent cAMP and βarrestin2 recruitment versus oliceridine concentration. Blue arrows indicate therapeutic range of blood concentrations of morphine (0.04-0.16 μM) and oliceridine (0.12-0.31 μM) (**supplementary table 1**). Red arrows indicate range of βarrestin2 recruitment for morphine and oliceridine corresponding to the tested therapeutic range. 'Δ' is the βarrestin2 recruitment difference between morphine and oliceridine at the maximum of the therapeutic range. No difference is discernable at the low end of the therapeutic range and only a small difference at the high end of the therapeutic range. Doses giving a larger blood concentration (e.g. log M = -5) of each would be expected to yield significantly different βarrestin2 recruitment.

Supplementary References

Buschmann, H., Winter, W., Graudums, I., and Jansen, P., inventor(s); Gruenenthal GmbH (Aachen, DE), assignee(s). *Method of separating the racemate of tramadol. United States Patent 5,723,668* (1998a).

Buschmann, H., Winter, W., Graudums, I., Jansen, P., Strassburger, W.W.A., and Friederichs, E.J., inventor(s); Gruenenthal GmbH (Aachen, DE), assignee(s). *Method of preparing the enantiomers of O-dimethyltramadol. United States Patent 5,728,885* (1998b).

Collins, S.L., Faura, C.C., Moore, R.A., and Mcquay, H.J. (1998). Peak plasma concentrations after oral morphine: a systematic review. *J Pain Symptom Manage* 16**,** 388-402. doi.

Dewire, S.M., Yamashita, D.S., Rominger, D.H., Liu, G., Cowan, C.L., Graczyk, T.M., Chen, X.T., Pitis, P.M., Gotchev, D., Yuan, C., Koblish, M., Lark, M.W., and Violin, J.D. (2013). A G protein-biased ligand at the mu-opioid receptor is potently analgesic with reduced gastrointestinal and respiratory dysfunction compared with morphine. *J Pharmacol Exp Ther* 344**,** 708-717. doi: 10.1124/jpet.112.201616.

Evans, G.R. (2001a). Highly efficient resolution of (±)-tramadol with di-p-toluoyl-tartaric acid (DTTA). *Tetrahedron: Asymmetry* 12**,** 1663-1670. doi.

Evans, G.R., inventor(s); Darwin Discovery, Ltd. (Great Britain), assignee(s). *Process. United States Patent 6,323,368* (2001b).

Gillen, C., Haurand, M., Kobelt, D.J., and Wnendt, S. (2000). Affinity, potency and efficacy of tramadol and its metabolites at the cloned human mu-opioid receptor. *Naunyn Schmiedebergs Arch Pharmacol* 362**,** 116-121. doi.

Grond, S., and Sablotzki, A. (2004). Clinical pharmacology of tramadol. *Clin Pharmacokinet* 43**,** 879-923. doi: 10.2165/00003088-200443130-00004.

Leow, K.P., Smith, M.T., Williams, B., and Cramond, T. (1992). Single-dose and steady-state pharmacokinetics and pharmacodynamics of oxycodone in patients with cancer. *Clin Pharmacol Ther* 52**,** 487-495. doi: 10.1038/clpt.1992.176.

Rauck, R.L., Oh, D.A., Singla, N., Koch, C., Parikh, N., Nalamachu, S., Wilson, D., Yu, J., and Vetticaden, S. (2017). Pharmacokinetics and safety of fentanyl sublingual spray and fentanyl citrate intravenous: a multiple ascending dose study in opioid-naive healthy volunteers. *Curr Med Res Opin* 33**,** 1921-1933. doi: 10.1080/03007995.2017.1371681.

Senanayake, C.H., Jerussi, T.P., Grover, P.T., Fang, Q.K., and Currie, M., inventor(s); Sepracor Inc. (Marlborough, MA), assignee(s). *Tramadol analogs and uses thereof. United States Patent 6,780,891* (2004).

Soergel, D.G., Subach, R.A., Burnham, N., Lark, M.W., James, I.E., Sadler, B.M., Skobieranda, F., Violin, J.D., and Webster, L.R. (2014). Biased agonism of the mu-opioid receptor by TRV130 increases analgesia and reduces on-target adverse effects versus morphine: A randomized, double-blind, placebo-controlled, crossover study in healthy volunteers. *Pain* 155**,** 1829-1835. doi: 10.1016/j.pain.2014.06.011.

Winpenny, D., Clark, M., and Cawkill, D. (2016). Biased ligand quantification in drug discovery: from theory to high throughput screening to identify new biased mu opioid receptor agonists. *Br J Pharmacol* 173**,** 1393-1403. doi: 10.1111/bph.13441.

Zebala, J.A., inventor(s); Syntrix Biosystems, Inc., assignee(s). *Compositions for overcoming resistance to tramadol. Patent 9,717,701* (2017).

Zebala, J.A., Searle, S.L., Webster, L.R., Johnson, M.S., Schuler, A.D., Maeda, D.Y., and Kahn, S.J. (2019). Desmetramadol has the safety and analgesic profile of tramadol without its metabolic liabilities: consecutive randomized, double-blind, placebo- and active comparator-controlled trials. *J Pain* 20**,** 1218-1235. doi: 10.1016/j.jpain.2019.04.005.
